# P53 Bacteria in the Uganda Cancer Institute ward environment—potential reservoirs for MDR organisms

**DOI:** 10.1093/jacamr/dlaf118.060

**Published:** 2025-07-14

**Authors:** Margaret Lubwama, Lesley Hoyles, Simon Sekyanzi, Benon Asiimwe, George Katende, Jody Winter

**Affiliations:** Department of Medical Microbiology, College of Health Sciences, Makerere University, Kampala, Uganda; Department of Biosciences, Nottingham Trent University, Nottingham, UK; Uganda Cancer Institute, Kampala, Uganda; Department of Medical Microbiology, College of Health Sciences, Makerere University, Kampala, Uganda; Department of Medical Microbiology, College of Health Sciences, Makerere University, Kampala, Uganda; Department of Biosciences, Nottingham Trent University, Nottingham, UK

## Abstract

**Background:**

Infections caused by MDR bacteria are one of the main dose-limiting toxicities among patients with cancer receiving chemotherapy and are associated with high mortality. At the Uganda Cancer Institute (UCI) we have previously demonstrated high rates of bacteraemia caused by MDR bacteria. However, it is unclear whether such infections are caused by gastrointestinal carriage or transmission between individuals.

**Objectives:**

To evaluate if the ward environment at the UCI could be a potential source of MDR bacteria causing infections in patients with cancer.

**Methods:**

We mapped out the lymphoma treatment centre (LTC) and the paediatrics wards at the UCI. Swabs were obtained from inanimate environment surfaces including stethoscopes, blood pressure cuffs, ward door entrances, bed rails, ward tables, bathroom floors, sinks, toilet handles and toilet bowls. The swabs were plated onto blood agar and MacConkey agar and incubated under aerobic conditions. Purity plates were made from the colonies that grew. Identification of bacteria was carried out using biochemical methods, BD Phoenix, and/or MALDI-TOF MS. Antimicrobial susceptibility tests were carried out using the Kirby-Bauer disc diffusion method and interpreted according to CLSI guidelines.

**Results:**

A total of 92 bacterial isolates were characterized. Most of the isolates were Gram-negative bacteria (*n*=73, 79%). Among the Gram-positive bacteria, 12/19 (63%) were *Bacillus spp.*, 5/19 (26%) were coagulase negative staphylococcus, and 2/19 (11%) were *Enterococcus* spp. (*Enterococcus hirae*, and *Enterococcus casseliflavus*). Among the Gram-negative bacteria, 25/73 (34%) were *Klebsiella pneumoniae*, 20/73 (27%) were *Acinetobacter* spp., 16/73 (22%) were *Citrobacter* spp., 9/73 (12%) were *Escherichia coli*, and 1/73 (1%) were *Enterobacter* spp., *Proteus mirabilis* and *Pseudomonas aeruginosa* each. Among the Enterobacterales, resistance rates were highest for amoxicillin/clavulanic acid (46/52, 89%), ceftriaxone (34/52, 65%), ceftazidime (29/52, 56%), ciprofloxacin (37/52, 71%) and trimethoprim/sulfamethoxazole (32/52, 62%). Thirty-three (63%) of the Enterobacterales and 5/21 (24%) of the non-Enterobacterales were MDR. The two *Enterococcus* spp. were non-susceptible to vancomycin. The toilet bowl swabs (*n*=6) each yielded at least one MDR organism. Bacteria with similar phenotypic characteristics and antimicrobial susceptibility patterns were observed in the paediatric ward.

**Conclusions:**

There is a high prevalence of MDR bacteria isolated from inanimate ward environments at UCI. Isolates from the toilet bowls could reflect gastrointestinal colonization of patients as a source of MDR bacteria which could spread from one patient to another. Observation of MDR bacteria with similar phenotypic characteristics and antimicrobial susceptibility patterns at distinct locations may indicate the possibility of transmission within the wards.

**Next steps:**

Using WGS, we will determine the relationships between the bacteria isolated from the environment and the bacteria isolated from both blood and gastrointestinal tract of patients with cancer and bacteraemia. This will provide valuable information for the development of informed infection prevention and control guidelines and policies at the UCI.

